# The effectiveness of an interactive organ donation education intervention for Dutch lower-educated students: a cluster randomized controlled trial

**DOI:** 10.1186/s13063-019-3882-6

**Published:** 2019-11-21

**Authors:** Esther Steenaart, Rik Crutzen, Math J. J. M. Candel, Nanne K. de Vries

**Affiliations:** 1 Department of Health Promotion, CAPHRI Maastricht University, PO Box 616, 6200 MD Maastricht, The Netherlands; 2 Department of Methodology and Statistics, CAPHRI Maastricht University, PO Box 616, 6200 MD Maastricht, The Netherlands

**Keywords:** Education program, Lower education, Vocational education, Adolescents, Organ donation registration, The Netherlands

## Abstract

**Background:**

Organ donation registration rates remain low, especially among people with lower educational levels. An interactive educational intervention was developed to prepare lower-educated students in the Netherlands for making a well-informed decision about organ donation. This article reports on the effects of this intervention on the intention to register (i.e., the primary outcome in the study at hand) and beliefs regarding organ donation.

**Materials and methods:**

The effectiveness was investigated in a post-test-only cluster randomized controlled trial, in which the intervention was offered to the experimental group and after measurement also to the control group. Randomization to the experimental and control groups took place at a class level. Teachers of schools for Intermediate Vocational Education who taught a course on Citizenship delivered three intervention elements (i.e., video fragments and discussion, quizzes with tailored feedback and exercise filling out a registration form) to their students during two 50-min lessons.

**Results:**

A total of 1170 students participated in the trial and filled out a questionnaire (45 experimental and 43 control classes). Compared to the control group, students in the experimental group had higher odds of having positive registration intentions (OR = 1.81; 95%CI [1.10–2.96]), their perceived knowledge was higher (B = 0.62; 95%CI [0.23–1.01]) and they had higher intentions to talk to family members (B = 0.68; 95%CI [0.28–1.08]) and friends (B = 0.36; 95%CI [0.07–0.66]) about organ donation. There were no effects on the choice students intended to register (OR = 1.08; 95%CI [0.67–1.73]).

**Conclusions:**

Providing education in a classroom setting is an effective tool in increasing registration intentions. Despite uncertainties about the effects on actual registration behavior, a larger-scale dissemination of this intervention is recommended. Providing clear information and opening the discussion about organ donation is an important and promising first step towards higher registration rates.

**Trial registration:**

The Dutch Trial Register, ID: NTR6771. Registered on 24 October 2017. https://www.trialregister.nl/trial/6557

## Introduction

Despite many efforts to increase donor rates in the Netherlands, the high demands for organs and tissues are not met. An important reason is that registration numbers remain low. The Dutch registration system allows persons to register several options, among which also registration as a non-donor. Only 42% of the Dutch population has registered their preference regarding organ donation, of which 58% registered as a donor [1]. This results in long waiting lists [2], but also places a high burden on family members and medical professionals [3–5]. In case someone dies without being registered, family members are asked for consent. This is often an overwhelming experience in sad circumstances [5]. Moreover, medical professionals expressed having difficulties talking to families about organ donation [3]. Up to 10% of the families are not even approached to ask for consent [4]. This all leads to a high refusal rate (67%) in cases where no consent or objection is registered [5].

Raising awareness about organ donation (registration) and encouraging people to make a decision is important to increase the registration rates. An important strategy, designed to reach abovementioned goals, is aimed at youngsters who come of age. Every year, the Dutch Ministry of Health, Welfare and Sports sends a letter to adolescents who just turned 18 years asking them to register a decision. This leads to a response rate of about one in three (including the people who already registered before receiving that letter) [6]. This shows that the majority of the 18-year-olds has not made a decision yet or did not register this. Studies show that there are many false beliefs about organ donation that could stand in the way of informed decision-making [7]. Youngsters should be supported in their decision process in order to increase the number of registrations.

Especially lower-educated adolescents could benefit from this support as a lower educational level is associated with lower registration rates [8]. Moreover, low literacy is a serious problem among this group [9], leading to difficulties with understanding written information about health and filling out forms [10]. Filling out a registration form and understanding and processing all information in the accompanied letter are, therefore, challenging. Therefore, we developed an educational program to prepare lower-educated students in the Netherlands for a well-informed decision about organ donation. It concerns an adapted version of an existing program aimed at high-school students, which was already proven to be effective [11]. The original program was based on the concepts of the Social Cognitive Theory extended with factors from other theoretical perspectives. A recent determinants study among lower-educated adolescents was used as an input to tailor the program to a lower-educated target group [7]. Disseminating this adapted version to lower-educated students through schools for Intermediate Vocational Education (IVE) could help in reaching a larger group of adolescents and possibly increase the number of registrations. However, prior to dissemination it is important to study the program’s effectiveness. This will be done in a cluster randomized controlled trial (CRCT) to evaluate the effects of the intervention on beliefs regarding organ donation and the intention to register in classes that received the program compared to classes that did not. This effect evaluation is combined with a process evaluation. This article focuses on the effectiveness of the intervention; the results of the process evaluation will be reported elsewhere.

## Materials and methods

### Aims

The effect evaluation provides insight into the effectiveness of the intervention on beliefs regarding organ donation and the intention to register (as donor or non-donor).

### Design

**Fig. 1:**
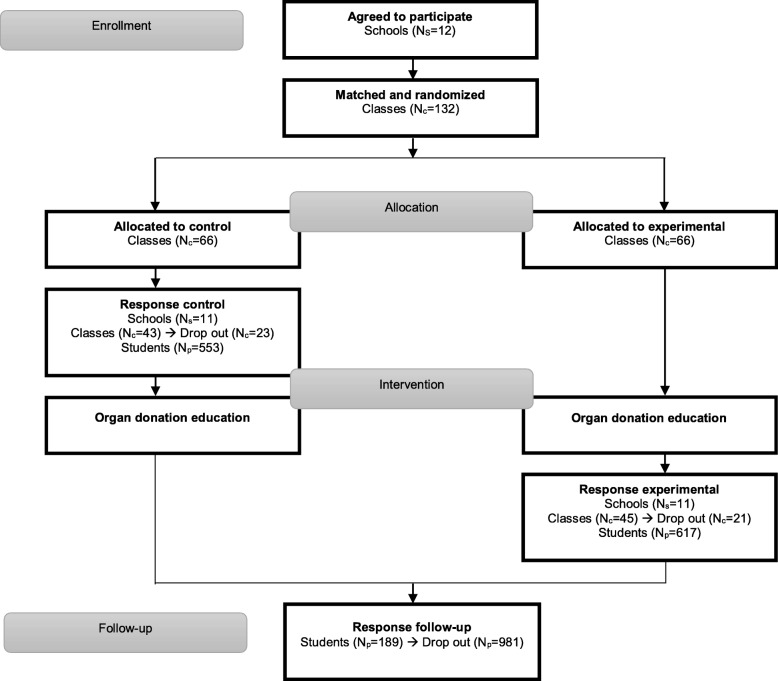
Flowchart of the participants

This article adhered to the Consolidated Standards of Reporting Trials (CONSORT) 2010 Checklist of information to include when reporting a cluster randomized trial (see Additional file 1 and Fig. 1). The effectiveness of the intervention was investigated in a post-test-only CRTC, in which the intervention was offered to the experimental group and, after measurement, also to the control group. This design was most suitable in a school setting to ensure that all students would receive the intervention. Participants in the control group filled in the evaluation questionnaire just before receiving the intervention, while participants in the experimental groups did this at the end of the second lesson. Randomization to the experimental and control groups took place at the class level. More details on the content of the intervention, study design and methodology can be found in the protocol article [12].

### Recruitment and procedure

Recruitment started in September 2017 and stopped in June 2018, as the summer vacation started in July. Teachers of Dutch schools for IVE who taught a course on Citizenship were invited by e-mail to use the intervention and participate in the study. Different methods were used to identify these teachers, including the use of contact persons from earlier studies in our department (not focused on organ donation, but other health-related topics), recruitment via key persons from a national IVE organization and using e-mail addresses that were provided on the schools’ websites. Region and study discipline (Health and Well-Being, Engineering, Economic, Social Services, Green and Security) were taken into account to ensure a good representation of the target group. Within IVE, there are four educational levels (where 4 is the highest level), but these are all considered low as opposed to college or university level. On class level, only students from levels 2 to 4 were included. Students in level 1 (also known as entry training) did not graduate from high school and have a large variety in cognitive abilities. Therefore, we cannot guarantee that they will be able to participate properly in the program. No additional inclusion or exclusion criteria were set as students were recruited via their teachers. Students were typically in the age group 15 years to early 20s, but older students were no exception nor excluded.

Please provide replacement figure file. Otherwise, kindly advice if we can retain the current presentation.Teachers who accepted the invitation were asked to draw up an inventory of the number of classes for participation, either being taught by themselves or by colleagues. This led to the inclusion of 132 classes in 12 schools. Classes were matched by a member of the study team to improve equivalence of the groups. Classes were (where possible) matched on study discipline and educational level, and matched classes were from the same school. From each pair of matched classes, one class was randomly assigned to the experimental condition by computer software, while the other class was then assigned to the control condition (*N* = 66 classes in the experimental group, *N* = 66 classes in the control group) (Fig. 1).

Teachers received a personalized link via e-mail to reach the website with the educational program. This link was used by both the teacher and the students of that class. Further, they received the questionnaires, instructions and a manual in paper form. Teachers were instructed to either distribute the questionnaires prior to delivering the intervention (control groups) or the other way around (experimental groups). The control classes completed the questionnaires before the first lesson. All participants then received the intervention, taught by their own teachers. The intervention consisted of two 50-min lessons addressing three elements. The first lesson focused on increasing involvement, encouraging positive beliefs and counterbalancing negative beliefs. This was done by watching video fragments followed by plenary discussions. In the second lesson, students first received tailored feedback on misconceptions they might have had by individually working with two quizzes on the website. Moreover, they did an exercise on filling in a simulated organ donation registration form to enhance their self-efficacy. Immediately after the intervention was delivered, students in the experimental classes filled out the questionnaires.

Teachers were asked to return the forms afterwards. Not all forms were returned, leading to a drop-out of *N* = 44 classes, of which one was an entire school (with *N* = 22 classes) (Fig. 1). Drop-out was due to problems with planning the lessons or staff turnover, leaving *N* = 88 classes (*N* = 45 in the experimental group, 617 students, *N* = 43 in the control group, 553 students).

Six months to 1 year after implementation, teachers were contacted for a follow-up measurement. This entailed an online questionnaire for students who received the intervention. We decided not to recruit new control groups (while this was indicated in the protocol article [12]), as this appeared unfeasible. Instead, we compared registration rates of the follow-up measurement with the original control group. The follow-up measurement led to a response of *N* = 189 students. High drop-out rates were found because of multiple reasons such as students graduating the year before, being spread over new classes, and conducting an internship (and, therefore, not being present at school).

### Ethical approval

The study was approved by the Ethics Committee of the Faculty of Health, Medicine and Life Sciences, Maastricht University, on 23 October 2017 (reference number: Steenaart/231017) and registered at the Dutch Trial Register (NTR6771; https://www.trialregister.nl/trial/6557). Students provided informed consent (after randomization) after explanations about the study, the opportunity to withdraw their consent at any time, and the anonymous way in which their data would be processed. They were provided with contact information in case they had any further questions.

### Measurements

The questionnaire in this study was largely based on the questionnaire used in a preparatory study [7]. The questionnaire assessed demographics, registration behavior, registration intention and beliefs regarding organ donation. The full questionnaire is available at https://osf.io/2jf7v/?view_only=4ed46d09a1874184a17636ebbd019415.

#### Demographics

Demographic variables included sex, age, study discipline, study level, country of origin and religion. Country of origin of the student, mother and father were assessed and combined into one item; having a western or non-western background. Students for whom one or more parents were from a non-western country, or who were born in a non-western country themselves, were labeled as non-western, while students from whom both parents were from a western country and who were born in a western country themselves were labeled as western [13]. Religion was also dichotomized into being religious or not.

#### Registration behavior and intention

Participants were asked whether they had already registered a decision regarding organ donation (yes/no/don’t know) and, if applicable, what choice they registered (posthumous organ and tissue donor/posthumous donor for specific organs and tissues/non-donor/leave the decision to the next of kin/leave the decision to a specific person). Unregistered participants were then asked whether they intended to register a decision (yes/no/don’t know) and, if applicable, the choice they would like to register (see the abovementioned options). The intention to register was the primary outcome measurement. All registration-related questions were dichotomized. Registration status was dichotomized into registered (yes) and not registered (no/don’t know), while registration choice was dichotomized into being a donor (posthumous organ and tissue donor/posthumous donor for specific organs and tissues) and not being a donor (non-donor/leave the decision to the next of kin or leave the decision to a specific person/don’t know). The intention questions were dichotomized in the same manner.

#### Beliefs regarding organ donation

The beliefs regarding organ donation included items related to attitude, self-efficacy, knowledge and social outcomes. The questionnaire contained 25 questions on beliefs, all answered on a 7-point Likert scale (totally disagree to totally agree). These questions were based on constructs from over 30 (mainly qualitative) existing studies and have been used before [7].

The 1-year follow-up measurement consisted of a short online questionnaire, primarily assessing registration status (yes/no/I don’t know) and if applicable, the choice they registered (see the earlier-mentioned options). However, as the response rate at follow-up was very low (*N* = 189) and over 50% of these participants did not remember receiving the program, the results of this follow-up measurement will not be reported, as no valid conclusions can be drawn from this.

### Statistical analyses

**Fig. 2:**
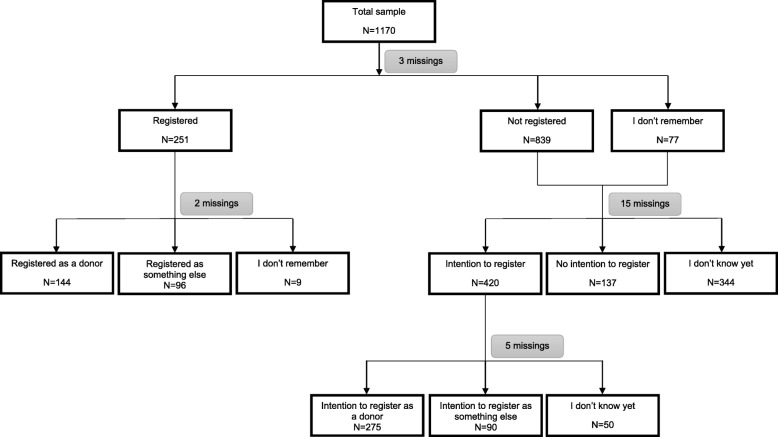
Flowchart regarding the outcomes

All data were analyzed using SPSS 24 and a two-sided *P* value ≤ .05 was considered significant for all analyses. The first aim of this study was to investigate the effectiveness of the intervention on the intention to register a decision regarding organ donation (i.e., the primary outcome in the study at hand). These analyses were only done among people who were not registered yet or did not remember (*N* = 916) (Fig. 2). Secondary outcomes were the choice that students intend to make and beliefs regarding organ donation. Since students were nested within classes within schools, multilevel analyses were used to examine the relationship between the intervention and (determinants of) organ donation registration intentions. For each analysis a step-by-step procedure was used to find the best model fit, starting with the most complex model (unstructured). The structure of the random-effect model was simplified where needed, by first removing the random slope and then the random intercept at the class level and/or school level. For more details about each model, the full syntax that was used can be found on OSF: https://osf.io/2jf7v/?view_only=4ed46d09a1874184a17636ebbd019415.

The effects of the intervention on the primary outcome were examined using multilevel nominal regression analyses, in which intervention group (control/experimental) was the main independent variable. Two different analyses were run; one for the dichotomized outcome (yes/no) and one for the original outcome (yes/no/don’t know). Both analyses were adjusted for several demographic characteristics (i.e., sex, age, educational level, religion, migration background and having had organ donation education before) as they were expected to be associated with the outcomes. To identify possible interaction effects, interaction terms of age, sex and educational level with the intervention variable were separately added to the analysis model in case of the dichotomized outcome (as this was our primary focus).

The effects of the intervention on the choice that students intended to make was done in a similar analysis. However, only the dichotomized outcome (donor/non-donor) was used, as some categories of the original outcome were chosen by too few students (e.g., I want to leave the decision to a specific person was chosen by only three students) to allow for reliable analyses. The model was adjusted for the same demographic characteristics as were used for the primary outcome.

Differences in beliefs regarding organ donation were examined with multilevel linear regression analyses, in which the intervention group (control/experimental) was again the main independent variable. A total of 25 analyses were run, one for each belief separately. These models were again adjusted for the same set of demographic characteristics. Seven beliefs were strongly skewed to the right. When the distribution of continuous outcomes is not symmetric and the values are always positive, a gamma distribution could be a better representation than the normal distribution. Therefore, for these beliefs, a gamma regression model was used [14]. To control for multiple testing, the Benjamini-Hochberg procedure was used with a false discovery rate of 5% [15]. An Excel template was used to calculate the adjusted significance levels.

The data file, syntax and excel templates used are available at https://osf.io/2jf7v/?view_only=4ed46d09a1874184a17636ebbd019415. These efforts are taken to maximize scrutiny, foster accurate replication, and facilitate future data syntheses (e.g., meta-analyses) [16].

## Results

### Participants

Participants in the trial had a mean age of 18.05 years (standard deviation (SD) = 2.02) and 31.6% were male. Students from six different study disciplines were included, of which most were enrolled in a Health and Well-Being program (44.9%). Students from levels 4 (67.7%), 3 (19.5%) and 2 (12.8%) participated, which is representative of the population of IVE students in the Netherlands [17]. The majority was not religious (62.0%) and had a western background (81.6%). Students (or parents from students) born in 75 different countries participated in the evaluation study. About one in five students said that they had already registered a decision regarding organ donation (21.5%), of whom 57.9% registered as a donor (with or without restrictions). Of those who did not register yet, 46.6% had the intention to make a decision, of which 66.3% would like to register as a donor (Fig. 2). There were large differences in intention rates between schools, ranging from 32.9 to 60.9%, emphasizing the importance of using mixed models. An overview of the demographic characteristics of the study sample can be found in Table 1.

**Table 1:** Demographic information of participants

	Control condition (*N* = 553)	Experimental condition (*N* = 617)	Total (*N* = 1170)
Sex (male)	29.5%	33.8%	31.6%
Age	18.04 ± 2.06	18.05 ± 1.99	18.05 ± 2.02
Younger than 18 years	47.1%	48.9%	48.1%
18 years	24.9%	21.9%	23.3%
Older than 18 years	28.0%	29.2%	28.6%
Study discipline			
Health and Well-Being	50.8%	39.5%	44.9%
Engineering	7.6%	11.8%	9.8%
Economics	25.5%	31.4%	28.6%
Social Services	7.1%	7.6%	7.4%
Green	6.0%	6.3%	6.2%
Security	3.1%	3.2%	3.2%
Educational level			
Level 2	15.2%	10.7%	12.8%
Level 3	20.8%	18.3%	19.5%
Level 4	64.0%	71.0%	67.7%
Religion			
Not religious	59.0%	64.6%	62.0%
Islam	12.1%	9.5%	10.7%
Catholic	11.9%	10.9%	11.3%
Dutch Reformed	2.9%	3.3%	3.1%
Calvinist	3.5%	4.3%	3.9%
Jewish	0.5%	0.3%	0.4%
Other	10.1%	7.1%	8.5%
Migration background (western)	79.7%	83.4%	81.6%
Registration status			
Not registered	73.3%	70.6%	71.9%
Registered	18.9%	23.9%	21.5%
Don’t know	7.8%	5.5%	6.6%
Registration choice			
Donor	31.1%	28.1%	29.3%
Restricted donor	24.3%	31.5%	28.5%
No donor	40.8%	26.0%	32.1%
Next of kin	1.0%	7.5%	4.8%
Specific person	1.0%	2.1%	1.6%
Don’t know	1.9%	4.8%	3.6%
Intention to register			
Yes	40.1%	52.8%	46.6%
No	18.1%	12.4%	15.2%
Don’t know	41.7%	34.8%	38.2%
Intention to become a donor			
Donor	32.2%	23.2%	27.0%
Restricted donor	33.3%	43.6%	39.3%
No donor	12.1%	21.2%	17.3%
Next of kin	5.2%	2.5%	3.6%
Specific person	1.7%	0.0%	0.7%
Don’t know	15.5%	9.5%	12.0%

Data presented as mean ± standard deviation (SD) for continuous variables and percentages for categorical variables

### Registration intentions

The intra-cluster coefficient (ICC) for registration intention was 0.10 at class level and 0.03 at school level [18]. Students in the experimental group had higher odds of having positive registration intentions, compared to students in the control group (OR = 1.81; 95%CI [1.10–2.96]) (Table 2). After adjusting for sex, age, educational level, migration background, religion and having had education about organ donation before, the effect of the intervention on registration intentions was comparable (OR = 1.74; 95%CI [1.11–2.73]) (Table 3). No interaction effects were found for sex, age and educational level (see Additional files 2, 3 and 4, respectively).

**Table 2:** Effect of organ donation education on students’ registration intentions

	Intention to register_dich_ (odds of yes versus no)		Intention to register (0 = no/1 = yes/2 = don’t know)				Intention to register as a donor_dich_ (odds of yes versus no)	
			(odds of yes versus no)		(odds of don’t know versus no)			
	OR (95%CI)	*P*	OR (95%CI)	*P*	OR (95%CI)	*P*	OR (95%CI)	*P*
Intervention group (experimental versus control)	**1.81 (1.10–2.96)**	**.02**	**1.93 (1.30–2.85)**	**.001**	1.22 (0.82–1.82)	.33	1.08 (0.67–1.73)	.76

*P* values < .05 are printed in bold

**Table 3:** Effect of organ donation education on students’ registration intentions, adjusted for demographic variables

Predictor	Intention to register_dich_ (odds of yes versus no)		Intention to register (0 = no/1 = yes/2 = don’t know)				Intention to register as a donor_dich_ (odds of yes versus no) (0 = no/1 = yes)	
			(odds of yes versus no)		(odds of don’t know versus no)			
	OR (95%CI)	*P*	OR (95%CI)	*P*	OR (95%CI)	*P*	OR (95%CI)	*P*
Intervention group (experimental versus control)	**1.74 (1.11–2.73)**	**.02**	**1.84 (1.18–2.85)**	**.01**	1.12 (0.72–1.73)	.61	1.10 (0.61–1.99)	.75
Sex (male versus female)	**0.64 (0.51–0.81)**	**< .001**	**0.31 (0.20–0.48)**	**< .001**	**0.41 (0.26–0.64)**	**< .001**	0.80 (0.52–1.24)	.31
Age								
18 versus < 18 years	0.81 (0.54–1.20)	.29	0.77 (0.45–1.31)	.33	0.87 (0.52–1.47)	.60	1.29 (0.71–2.34)	.41
> 18 versus < 18 years	**0.57 (0.39–0.83)**	**.003**	0.62 (0.35–1.08)	.09	0.95 (0.55–1.64)	.85	0.62 (0.56–1.54)	.86
Educational level								
level 3 versus level 2	**2.82 (1.64–4.84)**	**< .001**	**5.03 (2.38–10.61)**	**< .001**	**2.17 (1.09–4.30)**	**.03**	1.89 (0.89–4.01)	.10
level 4 versus level 2	**2.97 (2.18–4.04)**	**< .001**	**5.67 (3.08–10.43)**	**< .001**	**2.57 (1.50–4.40)**	**.001**	**2.80 (1.84–4.21)**	**< .001**
Religion (religious versus not religious)	1.13 (0.78–1.63)	.52	0.75 (0.46–1.24)	.26	**0.56 (0.34–0.93)**	**.03**	0.90 (0.59–1.36)	.61
Migration background (non-western versus western)	**0.46 (0.28–0.74)**	**.001**	**0.28 (0.16–0.50)**	**< .001**	0.61 (0.36–1.06)	.08	**0.45 (0.21–0.98)**	**.04**
Other organ donation education (yes versus no)	1.14 (0.91–1.42)	.26	0.98 (0.56–1.61)	.94	0.80 (0.48–1.33)	.39	**0.68 (0.49–0.94)**	**.02**

*P* values < .05 are printed in bold

A comparable effect was also found for the categorical outcome for registration intention (yes versus no and I don’t know versus no) when comparing having the intention to register to not having the intention to register (OR = 1.93, 95%CI [1.30–2.85]), but this was not significant when comparing students who did not know whether they intended to register to students not having the intention to register (OR = 1.22; 95%CI [0.82–1.82]). These effects were similar when adjustment was made for the demographic factors.

No significant effects were found of the intervention on the intention to register as a donor (versus as a non-donor) (OR = 1.08; 95%CI [0.67–1.73]). This was also the case after adjustment for demographic variables.

### Organ donation beliefs

In general, the intervention had small effects on beliefs regarding organ donation. Most scores on positive beliefs were a little higher in the experimental group compared to the control group, while scores on negative beliefs were lower. For 11 beliefs that were measured (12 after adjustment), no significant differences were found. See Table 4 for all (adjusted) regression coefficients. Important results are that students in the experimental group felt confident in having enough knowledge to make a decision about organ donation (B = 0.62; 95%CI [0.23–1.01]) and were more likely to talk to family members (B = 0.68; 95%CI [0.28–1.08]) and friends (B = 0.36; 95%CI [0.07–0.66]) about organ donation, compared to the control group. They also found it slightly more difficult to talk to family members (B = 0.08; 95%CI [0.01–0.16]) or friends (B = 0.13; 95%CI [0.04–0.23]), but the scores remained low (family 2.09 ± 1.6; friends 2.28 ± 1.72).

**Table 4:** Effect of organ donation education on students’ beliefs about organ donation

Outcome measurement	Control condition Mean ± SD	Experimental condition Mean ± SD	Regression weight of experimental (1) versus control (0)		Adjusted regression weight of experimental (1) versus control (0)	
			B (95%CI)	*P*	B (95%CI)	*P*
I have enough knowledge to make a decision about organ donation	4.53 ± 1.90	5.11 ± 1.61	0.62 (0.23– − 1.01)	**.002**^**a**^	0.57 (0.18–0.95)	**.004**^**a**^
I would like to know more about organ donation and registration	3.90 ± 1.81	3.57 ± 1.72	− 0.31 (− 0.48– − 0.14)	**< .001**^**a**^	− 0.36 (− 0.61– − 0.10)	**.01**^**a**^
I intend to talk about organ donation with family members	3.86 ± 1.98	4.47 ± 1.85	0.68 (0.28–1.08)	**.001**^**a**^	0.65 (0.27–1.02)	**.001**^**a**^
I intend to talk about organ donation with friends	3.27 ± 1.85	3.57 ± 1.91	0.36 (0.07–0.66)	**.02**^**a**^	0.33 (0.01–0.65)	**.04**
I find it hard to discuss organ donation with family members	1.91 ± 1.50	2.09 ± 1.61	0.08 (0.01–0.16)	**.02**	0.09 (0.00–0.18)	**.04**
I find it hard to discuss organ donation with friends	2.00 ± 1.56	2.28 ± 1.72	0.13 (0.04–0.23)	**.01**^**a**^	0.14 (0.05–0.23)	**.002**^**a**^
I rather not think about death	4.28 ± 2.18	4.41 ± 2.08	0.11 (− 0.07–0.28)	.23	0.07 (− 0.11–0.26)	.44
I still have enough time to register	4.98 ± 1.81	4.86 ± 1.92	− 0.17 (− 0.31– −.0.02)	**.02**	− 0.23 (− 0.45– − 0.01)	**.05**
The idea of my organs being in someone else’s body gives me a feeling of discomfort	3.46 ± 2.13	3.43 ± 2.08	− 0.04 (− 0.31–0.23)	.77	− 0.00 (− 0.28–0.27)	.98
Certain organs have an important value to me	3.24 ± 2.12	3.33 ± 2.08	0.11 (− 0.17–0.39)	.45	0.19 (− 0.13–0.50)	.24
When I die, I don’t want my organs to go to waste	3.94 ± 2.11	4.19 ± 2.09	0.26 (− 0.03–0.54)	.08	0.18 (− 0.10–0.46)	.21
After I die, my body needs to be intact	4.02 ± 2.03	4.18 ± 2.00	0.15 (− 0.12–0.43)	.28	0.21 (− 0.06–0.48)	.13
Some people are more deserving to receive my organ than others	4.17 ± 2.18	4.17 ± 2.13	− 0.03 (− 0.26–0.21)	.82	− 0.11 (− 0.41–0.18)	.45
If I register my decision, I prevent my family from having to make a difficult decision when I would die	5.26 ± 1.75	5.60 ± 1.62	0.13 (0.03–0.23)	**.01**^**a**^	0.12 (0.00–0.23)	**.05**
If I register my decision, medical professionals will honor my wishes	5.50 ± 1.63	5.86 ± 1.43	0.15 (0.04–0.26)	**.01**^**a**^	0.13 (0.05–0.21)	**.002**^**a**^
If I register my decision, I know what happens with my body when I would die	5.07 ± 1.88	5.36 ± 1.79	0.10 (0.01–0.20)	.08	0.11 (0.01–0.21)	**.03**
If I am an organ donor, I am happy I can help people in need	5.50 ± 1.68	5.70 ± 1.64	0.09 (0.01–0.17)	**.03**	0.06 (− 0.03–0.15)	**< .001**^**a**^
If I am an organ donor, I am afraid my family and friends see me as a deformed person because my organs were removed	2.70 ± 1.89	3.04 ± 1.98	0.12 (0.05–0.19)	**.001**^**a**^	0.13 (0.03–0.23)	**.01**
If I am an organ donor, I can find a sense of positive closure	4.42 ± 1.80	4.68 ± 1.75	0.26 (0.00–0.49)	**.03**	0.21 (− 0.02–0.43)	.07
If I am an organ donor, I run the risk of my organs being taken out before I died	3.20 ± 1.96	3.19 ± 1.90	0.05 (− 0.21–0.30)	.71	0.04 (− 0.25–0.32)	.79
If I am an organ donor, I am afraid my body will be mutilated	3.39 ± 1.98	3.52 ± 1.96	0.16 (− 0.12–0.43)	.26	0.19 (− 0.13–0.51)	.24
If I am an organ donor, medical professionals will choose the life of a patient who needs an organ over mine	3.46 ± 1.85	3.26 ± 1.87	− 0.19 (− 0.37- -0.01)	**.04**	− 0.16 (− 0.37–0.04)	.12
If I am an organ donor, I am more deserving of receiving an organ in case I need one	4.07 ± 1.99	4.07 ± 2.01	− 0.00 (− 0.22–0.22)	.99	0.04 (− 0.14–0.23)	.63
If I am an organ donor, my organs will be allocated to patients in an ethical manner	4.93 ± 1.60	5.22 ± 1.58	0.29 (0.08–0.49)	**.01**^**a**^	0.26 (0.04–0.47)	**.02**
If I am an organ donor, I run the risk of being declared dead too soon	3.23 ± 1.97	3.17 ± 1.62	− 0.03 (− 0.25–0.19)	.78	− 0.03 (− 0.23–0.17)	.78

*P* values < .05 are printed in bold

^a^Significant *P* values after correction for multiple comparisons according to the Benjamini-Hochberg procedure [14]

### Exploratory results

Table 3 shows some additional interesting findings. However, as no hypotheses were formulated for these analyses, the results are purely exploratory. Female students (OR = 0.64; 95%CI [0.51–0.81) and students with a western background (OR = 0.46; 95%CI [0.28–0.74]) had higher odds of having a positive registration intention, and so did students younger than 18 years of age (OR = 0.57; 95%CI [0.39–0.83] (compared to students older than 18 years of age). Also, differences can be observed in terms of educational level. When compared to level-2 students, students in level 4 (OR = 2.82; [1.64–4.84]) and level 3 (OR = 2.97; 95%CI [0.28–0.74]) had higher odds of having the intention to register a decision.

## Discussion

This paper describes the evaluation of a web-based and classroom-based organ donation education program aiming to encourage lower-educated adolescents to register their preference regarding organ donation. Compared to the control group, more students in the experimental group were intending to register a decision, their perceived knowledge was higher and they expressed greater intentions to talk to others about organ donation. There were no effects of the intervention on the intention to register as an organ donor. These effects are in line with our expectations as the purpose of the intervention was to support decision-making, not to convince students to register as organ donors. Moreover, no adverse effects of the intervention were found on the beliefs or uncertainty regarding their intentions. Finally, sex, age, educational level and migration background are possibly related to students’ intention to register a decision. These results are exploratory in nature and could be used for developing research questions for future studies.

The intervention evaluated in this study concerns an updated and simplified version of an intervention developed and evaluated by Reubsaet and colleagues [11]. The results of the present study are comparable to those of the first version of the intervention, where an increase in registration intention of 33% was found (compared to 32% in the present study). This suggests that organ donation education, when carefully adapted to a specific target group, has the potential to increase registration rates. Moreover, as no significant interactions were found between sex, educational level, age on the one hand and intervention condition on the other, the intervention seems suitable for a wide range of lower-educated students.

As only small differences were found on the beliefs in this study, this might indicate areas in which the intervention can improve further. However, a preparatory determinants study already showed that none of the beliefs are decisive individually and decision-making about organ donation is far more complex [7]. This seems a possible explanation for the findings in this study as the effects on beliefs are small, but a relatively large difference was found in registration intention. This suggests that the effects of the intervention might not be mediated by the explicit beliefs in the questionnaire, but by more complex processes.

Several other studies have shown that organ donation education can be effective in increasing knowledge [19–22], willingness/intention to register [19–22] and willingness to talk to family members among adolescents [19, 20, 23]. In that sense, the results of this study are in line with previous work. However, organ donation education is usually provided in a high-school setting or among students in higher levels of secondary education, without paying attention to lower educational levels (and also lower literacy levels). Especially with a sensitive and difficult topic like organ donation, a one-size-fits-all approach might not work. Even within this study among IVE students, teachers emphasized that students from level 2 and level 4 cannot be compared and should be treated differently [24]. Preliminary results of the process evaluation also showed that teachers made on-the-spot adaptations when delivering the intervention. It is, therefore, important to recognize that not all students are the same and organ donation education should be tailored to match students’ abilities.

There were some difficulties regarding the follow-up measurement. As registration records are private, we had to rely on self-reported registration behavior. The response on this follow-up measurement was low. This makes the effects of the intervention on registration behavior doubtful. A systematic review on classroom organ donation education found that none of the 15 included studies measured actual registration behavior [25]. The attempt we made in the current study shows the difficulties with assessing actual registration behavior and thus still leaves us with uncertainties about a possible intention-behavior gap regarding organ donation registration.

The number of participants during the intervention was also lower than planned (see the protocol article for the sample size calculation [12]). However, a very conservative estimation of the effect on intention to register was used in this calculation (i.e., a relative increase of 20%), while the actual effects of the original version of the intervention (33%) and the version used in this study (32%), are much higher. Moreover, we still managed to acquire a representative sample with a high diversity of geographical areas, study disciplines, educational levels and migration backgrounds.

### New donor law

In February 2018, a new donor law was approved by the Dutch Senate. This law, to be implemented in the summer of 2020, entails a variation on an opt-out system instead of the current opt-in system. Everyone aged over 18 years, who has not made a decision yet, will receive a letter asking them to register a decision. Failure to respond to this letter, or the reminder that will be sent 6 weeks later, results in a “no objection” registration in the Donor Registry. With this upcoming law change, the need for education increases [26].

The main goal of this law is to have more people make an explicit decision, but many people are not ready for this, do not know enough about the topic or might not understand how the new law works. This could potentially lead to many people with a “no objection” registration, instead of an explicit “yes” or “no,”, in which cases the decision will still come down to the relatives. Supporting people in the decision-making process is, therefore, crucial, as well as encouraging people to talk to family members. Studies have shown that relatives feel more confident in making a decision when they know what the deceased person wanted [27]. If not, they usually feel hesitant and indecisive, which in many cases leads to refusal [4]. So, even if adolescents are not ready to make a decision yet, talking about it at home should be encouraged. Students in this study expressed the view that they did not find it difficult to talk to family members or friends, but this might not translate into actually discussing donorship at home. Tamburlin and colleagues found that this can be very difficult, especially starting the conversation [23]. The intervention could benefit from more practical guidance in this.

The vote about the new donor law in February 2018 has been preceded by both public and political debates and has received a lot of media attention. As a response to this change in law, more people registered as a non-donor, especially more young people said “no” to organ donation in the last year [1]. This all happened during the trial period of the intervention and could, therefore, have influenced students’ attitudes towards organ donation registration.

## Conclusion

It is clear that supporting decision-making about organ donation is important, especially when it concerns lower-educated groups. This has been one of the first efforts to educate this underrepresented group. It became evident that providing education in a classroom setting is effective in increasing registration intentions. Despite uncertainties about the effects on actual registration behavior, a larger-scale dissemination of this intervention is recommended. As Cardénas and colleagues pointed out, decision-making regarding organ donation is very complex and is usually a process that evolves over time [21]. Providing clear information and opening the discussion about organ donation is an important and promising first step towards higher registration rates.

## Supplementary information

**Additional file 1.** Consolidated Standards of Reporting Trials (CONSORT) 2010 Checklist of information to include when reporting a cluster randomised trial.

**Additional file 2.** Effect of organ donation education on students’ intention to register, adjusted for demographic variables and intervention group*sex interaction.

**Additional file 3.** Effect of organ donation education on students’ intention to register, adjusted for demographic variables and intervention group*age interaction.

**Additional file 4.** Effect of organ donation education on students’ intention to register, adjusted for demographic variables and intervention group*educational level interaction.

## Data Availability

The dataset generated and analyzed during the current study is available on the Open Science Framework repository: https://osf.io/2jf7v/?view_only=4ed46d09a1874184a17636ebbd019415.

## Abbreviation

IVE: Intermediate Vocational Education
