# Supplementary material for: The effectiveness of an interactive organ donation education intervention for Dutch lower-educated students: a cluster randomized controlled trial
Source: Trials. 2019 Nov 21;20:643. doi: 10.1186/s13063-019-3882-6 (PMC6873467; doi:10.1186/s13063-019-3882-6)
Supplement: Supplementary file 2 — Additional file 2. Effect of organ donation education on students’ intention to register, adjusted for demographic variables and intervention group*sex interaction. [file 13063_2019_3882_MOESM2_ESM.docx]

Additional file 2: *Effect of organ donation education on students’ intention to register, adjusted for demographic variables and intervention group*sex interaction*

| Predictor | Intention to register_dich_  (odds of yes versus no) | |
| --- | --- | --- |
|  | OR (95% CI) | *P* |
| Intervention group  (experimental versus control) | **1.77 (1.17-2.69)** | **.01** |
| Sex (male versus female) | 0.66 (0.44-1.00) | .05 |
| Age | | |
| - 18 versus <18 | 0.81 (0.54-1.20) | .29 |
| - >18 versus <18 | **0.57 (0.39-0.83)** | **.003** |
| Educational level | | |
| - level 3 versus level 2 | **2.80 (1.60-4.91)** | **<.001** |
| - level 4 versus level 2 | **2.96 (2.15-4.08)** | **<.001** |
| Religion  (religious versus not religious) | 1.13 (0.78-1.63) | .52 |
| Migration background  (non-western versus western) | **0.46 (0.28-0.73)** | **.001** |
| Other organ donation education (yes versus no) | 1.14 (0.91-1.42) | .26 |
| Intervention group*Sex | 0.95 (0.59-1.54) | .84 |

P-values < .05 are printed in bold.
