# Supplementary material for: The effectiveness of an interactive organ donation education intervention for Dutch lower-educated students: a cluster randomized controlled trial
Source: Trials. 2019 Nov 21;20:643. doi: 10.1186/s13063-019-3882-6 (PMC6873467; doi:10.1186/s13063-019-3882-6)
Supplement: Supplementary file 3 — Additional file 3. Effect of organ donation education on students’ intention to register, adjusted for demographic variables and intervention group*age interaction. [file 13063_2019_3882_MOESM3_ESM.docx]

Additional file 3: *Effect of organ donation education on students’ intention to register, adjusted for demographic variables and intervention group*age interaction*

| Predictor | Intention to register_dich_  (odds of yes versus no) | |
| --- | --- | --- |
|  | OR (95% CI) | *P* |
| Intervention group  (experimental versus control) | 1.65 (0.93-2.91) | .09 |
| Sex (male versus female) | **0.65 (0.51-0.81)** | **<.001** |
| Age | | |
| - 18 versus <18 | 0.76 (0.44-1.30) | .32 |
| - >18 versus <18 | **0.53 (0.31-0.89)** | **.02** |
| Educational level | | |
| - level 3 versus level 2 | **2.82 (1.62-4.92)** | **<.001** |
| - level 4 versus level 2 | **2.99 (2.20-4.05)** | **<.001** |
| Religion  (religious versus not religious) | 1.12 (0.77-1.63) | .54 |
| Migration background  (non-western versus western) | **0.46 (0.28-0.74)** | **.002** |
| Other organ donation education (yes versus no) | 1.13 (0.89-1.43) | .33 |
| Intervention group*Age | | |
| - 18 versus <18 | 1.12 (0.60-2.08) | .72 |
| - >18 versus <18 | 1.18 (0.61-2.28) | .63 |

P-values < .05 are printed in bold.
