# Supplementary material for: The effectiveness of an interactive organ donation education intervention for Dutch lower-educated students: a cluster randomized controlled trial
Source: Trials. 2019 Nov 21;20:643. doi: 10.1186/s13063-019-3882-6 (PMC6873467; doi:10.1186/s13063-019-3882-6)
Supplement: Supplementary file 4 — Additional file 4. Effect of organ donation education on students’ intention to register, adjusted for demographic variables and intervention group*educational level interaction. [file 13063_2019_3882_MOESM4_ESM.docx]

Additional file 4: *Effect of organ donation education on students’ intention to register, adjusted for demographic variables and intervention group*educational level interaction*

| Predictor | Intention to register_dich_  (odds of yes versus no) | |
| --- | --- | --- |
|  | OR (95% CI) | *P* |
| Intervention group  (experimental versus control) | 2.50 (0.72-8.62) | .15 |
| Sex (male versus female) | **0.64 (0.50-0.81)** | **<.001** |
| Age | | |
| - 18 versus <18 | 0.80 (0.54-1.19) | .27 |
| - >18 versus <18 | **0.57 (0.40-0.83)** | **.004** |
| Educational level | | |
| - level 3 versus level 2 | **3.60 (2.14-6.07)** | **<.001** |
| - level 4 versus level 2 | **3.62 (2.00-6.56)** | **<.001** |
| Religion  (religious versus not religious) | 1.13 (0.78-1.63) | .53 |
| Migration background  (non-western versus western) | **0.46 (0.28-0.74)** | **.001** |
| Other organ donation education (yes versus no) | 1.14 (0.91-1.42) | .26 |
| Intervention group*Educational level | | |
| - level 3 versus level 2 | 0.62 (0.22-1.73) | .36 |
| - level 4 versus level 2 | 0.69 (0.20-2.41) | .56 |

P-values < .05 are printed in bold.
